# The possible role of cell cycle regulators in multistep process of HPV-associated cervical carcinoma

**DOI:** 10.1186/1472-6890-7-4

**Published:** 2007-05-24

**Authors:** Abeer A Bahnassy, Abdel Rahman N Zekri, Maha Saleh, Mohammad Lotayef, Manar Moneir, Osama Shawki

**Affiliations:** 1 Pathology Department, National Cancer Institute, Cairo University.1^st ^Kasr El-Aini st. Cairo, Egypt; 2 Virology and Immunology Unit, Cancer Biology Department, National Cancer Institute, Cairo University.1^st ^Kasr El-Aini st. Cairo, Egypt; 3 Clinical pathology department, National Cancer Institute, Cairo University.1^st ^Kasr El-Aini st. Cairo, Egypt; 4 Radiotherapy Department, National Cancer Institute, Cairo University.1^st ^Kasr El-Aini st. Cairo, Egypt; 5 Epidemiology and Biostatistics Department, National Cancer Institute, Cairo University.1^st ^Kasr El-Aini st. Cairo, Egypt; 6 Gynecology and Obstetrics Department, Kasr El-Aini School of Medicine, Cairo University, 1^st ^Kasr El-Aini st., Cairo, Egypt

## Abstract

**Background:**

Human papillomavirus (HPV) 16 and 18 are associated with cervical carcinogenesis through an interaction between HPV oncogenic proteins and cell cycle regulatory genes. However, the exact pathogenetic mechanisms are not determined yet.

**Methods:**

We investigated 43 invasive squamous cell carcinoma (ISCC), 38 CIN III, 11 CINII and 18 CINI for *cyclin D1, cyclin E, CDK4, p53, mdm-2, p21*^*waf*^*, p27*, *p16*^*INK*4*A*^, *Rb and Ki-67 *aberrations using immunohistochemistry and molecular techniques. Twenty samples of normal cervical tissues (NCT) were taken as a control.

**Results:**

There was a significant increase in the expression of *Ki-67*, *cyclin E, CDK4, p16*^*INK*4*A*^*, Rb (p= *0.003, 0.001, 0.001, 0.01) and a significant decrease in *p27*^*KIP*1 ^from NCT to ISCC (p = 0.003). Increased *cyclin D1, p21*^*waf*^*, p53, mdm-2 *expression, homozygous deletion (HZD) and promoter methylation (PM) of the *Rb *were detected in CINIII and ISCC only. On univariate analysis; tumor size, differentiation, lymph node status, FIGO stage, *Ki- 67, cyclin D1, p53 and p27*^*KIP*1 ^are significantly associated with reduced overall survival (OS) while on multivariate analysis; only FIGO stage, *Ki-67, cyclin D1, p53 and p27*^*KIP*1 ^were significant.

**Conclusion:**

1) Aberrations involving *p27*^*KIP*1^, *cyclin E, CDK4*, *p16*^*INK*4*A *^are considered early events in HPV 16 and 18-associated cervical carcinoma, whereas *cyclin D1 and p53 *pathway abnormalities are considered late events. 2) Immunohistochemical tests for *p16*^*INK*4*A*^*and cyclin E*, could help in early diagnosis of cervical carcinoma. 3) Only FIGO stage *p53, cyclin D1, p27*^*KIP*1 ^and *Ki-67 *are independent prognostic factors that might help in predicting outcome of cervical cancer patients.

## Background

Carcinoma of the uterine cervix emerges from a defined series of prenoplastic lesions with increasing cellular dysplasia referred to as cervical intraepithelial neoplasia (CIN) grade I, II and III [1]. A large body of knowledge supports the view that high risk HPV types (HR-HPV) are strongly associated with invasive squamous cell carcinoma (ISCC) and its precursors as they have the ability to transform normal cervical cells into neoplastic ones [2]. HPV 16 and 18 are the most commonly detected HR-HPV types in these lesions, and therefore their detection was proposed as a useful surrogate marker to diagnose cervical dysplasia and carcinoma in situ (CIS) [2,3].

Progress in cervical cancer research provided evidence that integration of HR-HPV DNA into the host cell genome results in elevated expression levels of the *E6 *and *E7 *proteins with subsequent interaction between these oncogenic proteins and the cell cycle machinery [2,4]. Expression of *E6 *and *E7 *proteins of HR-HPV types induces immortalization of cells through their inhibitory effects on the tumor suppressor proteins *pRb *and *p53*; respectively, altering the cell cycle control and leading to chromosomal instability [2]. Undermining of *pRb *growth-inhibitory role with release of *E2F *transcription factors render the cells independent of mitogenic stimuli [5], whereas inactivation of the *p53 *precludes its role as one of the major factors controlling cell proliferation since it arrests the cell cycle in response to DNA damage or direct the damaged cell to an apoptotic pathway. This function is abolished via *p53 *degradation by the *E6 *protein of oncogenic HPV types or via cytoplasmic sequestration by complexing to *mdm-2 *protein [6].

However the *E6-p53 and E7-Rb *model is not sufficient to inevitably produce cervical carcinoma although it has resulted in the identity of the viral gene's actions on numerous cellular proteins and processes normally involved in cellular growth and proliferation. This is evidenced by the spontaneous clearance of HPV infection and the long delay between the onset of persistent infection and the emergence of malignancy. Recent studies show an association between HR-HPV types and cell cycle regulators [2,7]. The cell cycle is governed by a family of *cyclins*, *cyclin dependent kinases (CDKs) *and their inhibitors *(CDKIs) *through activating and inactivating phosphorylation events. Attention has been focused on altered expression of G_1 _*cyclins *and *Cdks *because the major regulatory events leading to cell proliferation and differentiation occur within the G_1 _phase of the cell cycle. The D-type *cyclins *reach maximum levels of expression and form functional kinase complexes with *CDK4 *or *CDK6; *during the mid-G_1 _phase, whereas *cyclin-E *is expressed and associated with *Cdk2 *in an active complex near the G_1_-S boundary [5,8]. Active *CDK/cyclin *complex can be regulated by binding to *CDKI *(*p16*^*INK*4*A*^*, p21*^*waf*1 ^and *p27*^*KIP*1^) and inhibit cell cycle progression from G1 to S phase [7].

Deregulation of *p16*^*INK*4*A*^*, p21*^*waf*1 ^and *p27*^*KIP*1 ^has recently been reported in various human tumors. In HPV-associated cervical carcinoma, the expression and function of these proteins is supposed to be impaired by the action of viral oncopoteins *E6 *and *E7 *[9]. However, it is unclear how and when factors that are innate to the HPV-infected cells including genetic aberrations launch the host cell into an irreversible progression to cancer [7].

Therefore, we attempted to assess the contribution of *cyclin D1, cyclin E, CDK4, p53, mdm-2, p21*^*waf*^*, Rb, p27*^*KIP*1^*, p16*^*INK*4*A *^and *Ki-67 *expression to the development of HPV16/18-associated cervical carcinoma and to determine at what stage of carcinogenesis these aberrations start to manifest. The prognostic value of these aberrations was also investigated in relation to the standard clinicopathological prognostic factors and overall survival.

## Methods

### Clinical samples

The study included 110 fresh-frozen tissue samples that were selected from a total of 200 based on the positivity for HPV16 and/or18 and on the availability of clinical and follow-up data. The studied cases included 43 invasive squamous cell carcinoma (ISCC), 38 cervical intra-epithelial neoplasia (CIN) III, 11 CINII and 18 CINI cases were collected from patients who were diagnosed and treated at the hospitals of Kasr El-Aini School of Medicine and the National Cancer Institute (NCI), Cairo University during March 1999- July 2003 after a recent evidence of abnormal cervical cytology and/or conization biopsy (obtained by large loop excision of the transformation zone). Cases of ISCC were examined by two independent pathologists, classified and graded according to the World Health Organization (WHO) criteria and staged according to criteria of the International Federation of Gynecolology and Obstetrics (FIGO) [10]. The clinicopathological features of the studied cases are illustrated in table 1. Twenty normal cervical tissues (NCT) obtained from patients undergoing hysterectomy for medical conditions not related to the cervix were included in the study as a control. All control samples showed normal cervical cytology and histology and all were negative for HPV by PCR. In ISCC and CIN samples, only cases with ≥ 75% tumor cells were included in the study. A written consent was obtained from all patients prior to enrollment in the study, and the ethical committee of the NCI approved the protocol which was in accordance with the ethical guidelines of the 1975 Declaration of Helsinki.

### Cell lines

The HPV16 (*SiHa*), HPV18 (*HeLa*) positive cervical carcinoma and the mdm2 positive (SA-1) cell lines (from Institute of Human Genetics, Academic Medical Centre, Amsterdam, The Netherlands) were maintained in Dulbecco's Modified Eagle Medium (DMEM; Sigma, USA) supplemented with 10% fetal bovine serum (Sigma, USA) and used as controls.

### DNA extraction

High molecular weight DNA was extracted from fresh tumor and normal samples as well as cell lines, according to standard protocols [11].

### HPV detection and typing

The purified DNA was subjected to PCR amplification using general-purpose HPV primers (*GP5+ and GP6+) *which amplify conserved sequences in the HPV-L1 region (150 bp) as previously described [12]. PCR products were separated by electrophoresis on 1.5% agarose gel, transferred onto a nylon membrane (Hybond N +, Amersham) and analyzed using digoxigenin-labelled type-specific probes by chemiluminescent detection (ELOCA). The HPV probe consisted of a cocktail of 14 high-risk mucosal types [16, 18, 31, 33, 35, 45, 51, 52, 56, 58, 59, 62, 66, and 68] and two low-risk types [6 and 11].

### Immunohistochemistry

Tumors and normal tissue samples were put in 10% neutral buffered formalin and processed routinely for preparation of hematoxylin and eosin-stained slides and for immunohistochemical studies. Five micron thick sections were cut onto positive-charged slides and used for immunohistochemical detection of *cyclin D1, cyclin E, CDK4, p53, mdm-2, p21*^*waf*^,*p27*^*KIP*1^*, p16*^*INK*4*A*^*, Rb, and Ki-67*. The standard streptavidin-biotin-peroxidase detection technique was performed using the antibodies illustrated in table 2. Briefly, after deparaffinization in xylene and rehydration through graded alcohols, sections were microwaved in 0.01 M citrate buffer (pH 6.0) for 15 min. Endogenous peroxidase activity was blocked by immersion in 0.03% H_2_O_2 _in methyl alcohol for 30 min and 10% normal rabbit (for mouse primary antibodies) or goat serum (for rabbit primary antibodies) was applied to avoid non-specific reaction. Sections were then incubated with the primary antibodies or with non-immunized mouse or rabbit serum for the negative control at 4°C. After washing with PBS, biotinylated anti-muse or rabbit IgG was applied for 30 min at room temperature. The peroxidase-conjugated-streptavidin solution was applied for 30 min and visualized using 0.05% 3'-3' diaminobenziine (DAB). Counterstaining was performed with Meyer's hematoxylin [13].

**Figure 1:**
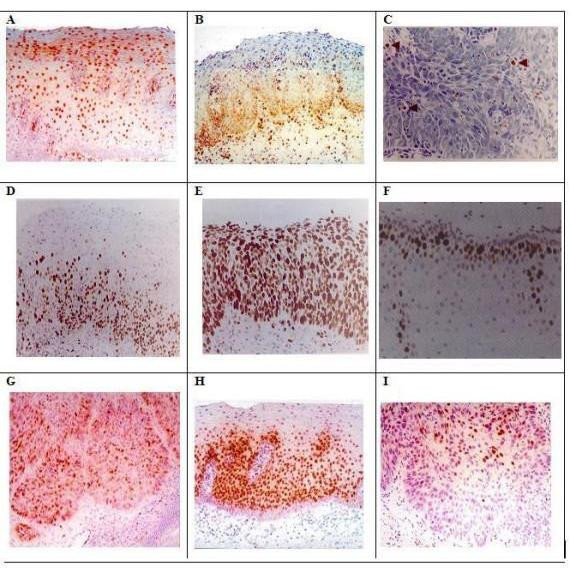
a) Positive immunostaining for *p27*^*KIP*1 ^*i*n the nuclei of normal cervical tissue, b) positive immunostaining for *p27*^*KIP*1 ^in CINI, c) lack of *p27*^*KIP*1 ^immunostaining in a case of invasive squamous cell carcinoma, d) positive immunostaining for *p16*^*INK*4*A *^in a case of CINII, e) positive immunostaining for *p16*^*INK*4*A *^in a case of III, f) negative nuclear imminostaining for *cyclinD1 *in normal cervical tissue except in the parabasal cells, g) a strongly positive immunostaining for *cyclinD1 *in a case of invasive squamous cell carcinoma, h) positive nuclear immunostaining for *Rb *in parabasal and intermediate squamous cells in hyperplastic squamous epithelium, and i) focal and weak nuclear staining for *Rb *in a case of invasive squamous cell carcinoma.

Only a distinct brown nuclear staining was scored positive. In NCT, cyclin *D1; cyclin E *and *CDK *were focaly expressed in the parabasal cells only, there was no immunostaining for *p53 and mdm-2*, *p21*^*waf *^was focally detected, *p27*^*KIP*1 ^was normally detected in > 50% of the cells and *Rb *was detected in all NCT samples. This was the normal expression pattern for the studied protein [8]. An aberrant expression of *cyclin D1, cyclin E, CDK4*, *p21*^*waf *^and *p16*^*INK*4*A *^was defined as staining in excess of normal tissues (> 20% for cyclin *D1; cyclin E, CDK4 *and > 10% for *p21*^*waf*^*and p16*^*INK*4*A*^) (Figure 1). *p53 *and *mdm-2 *overexpression was considered with > 10% positive cells. *L*oss of *p27*^*KIP*1 ^was defined as staining in < 50% of the cells and altered expression of *Rb *was defined as absence of nuclear staining in all sections examined [8]. Four semiquantitative classes were used to describe the percentage of positively-stained tumor cells: negative (no cells stained); +: minimaly positive (1–10% positive cells); ++: moderately positive (10–50% positive cells); and +++: markedly positive (> 50% positive cells). The positivity of each stain including *Ki-67 *was also described as a positivity index (PI) which indicated the number of positive cells in 1000 arbitrarily selected, manually counted cells at 200× magnification [4,6,13].

### Determination of p53 gene status

Exons 5–9 of the *p53 *gene were amplified separately using *p53 *Gene Analysis Kit (Visible Genetics Inc., Toronto, Canada) in all studied cases. Briefly, 3 ul of genomic DNA were added to a 25-μl PCR mixture according to manufacturer's instructions and then cycled through 35 cycles consisting of 94°C for 30 s, 60°C for 30 s, and 72°C for 60 s in a thermo-cycler (Perkin Elmer, Norwalk, CT, USA). PCR products were then visualized by electrophoresis in an ethidium bromide-stained 1% agarose gel. Single strand conformation polymorphism (SSCP) was performed as previously described [14]. All PCR samples with aberrant conformers on SSCP were sequenced using nested CY5/CY5.5-labeled primers provided in the *p53 *Gene Analysis Kit (Visible Genetics Inc., Toronto, Canada) according to supplier's instructions and analyzed using the MicroGene Blaster Automated DNA electrophoresis unit (Visible Genetics Inc., Toronto, Canada)

***Determination of cyclin D1, cyclin E and CDK4 gene status ***was performed using the differential PCR assay (DPA) as previously described with GAPDH as an internal control [15].

***Detection of ***DNA methylation in CpG islands of the *Rb *gene was done using a methylation specific PCR. Homozygous deletion (HZD) was assessed using a semiquantitative differential PCR as described by Nakamura et al. [16].

### Determination of p16 gene mutation

The presence of *p16 *gene mutations was assessed using the SSCP/sequencing according to Tripathi et al. [17].

***mdm-2 gene amplification ***was assessed using the primer sequence and PCR conditions of Oda et al [18] with phenyl alanine hydroxylase (PAH) as an internal control. The level of *mdm-2 *gene amplification was determined by comparing the intensities of the *mdm-2 *and PAH-PCR products for each of the samples with SA-1 cells as a positive control (seven folds). Samples showing more than 2 fold amplification were considered positive.

### Statistical methods

Statistical analysis was performed using the Stat View 4.5 software package (Abacus Concepts, Berkeley, CA). The Mann-Whitney non parametric test was used to compare the PIs of pairs of subjects and the Kruskal-Wallis test was used for categorical data. Correlation between the indexes was determined by a simple linear regression test. Kaplan-Meier method and the log-rank tests were used for the survival analysis. Univariate and multivariate Cox proportional hazards modeling were performed with overall survival (OS) as the end point. The median time of follow-up was 11.6 months for all patients and 33.5 months for survivors. Values of *p *< 0.05 were considered to be statistically significant.

## Results

### Ki-67 expression

**Figure 2:**
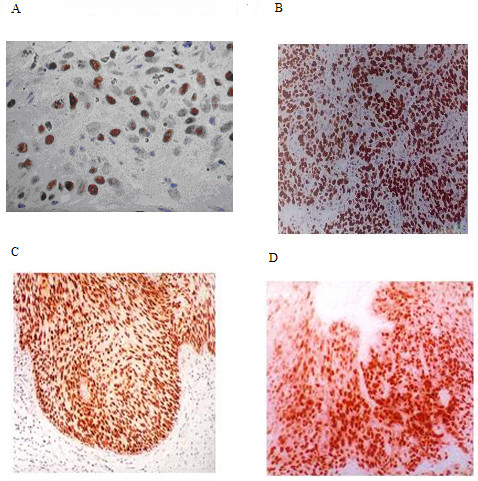
Cases of invasive squamous cell carcinoma showing a) strong nuclear immunostaining for *p53*, b) diffuse nuclear staining for *mdm2*, c) positive nuclear immunostaining for *p21*^*WAF *^and, d) positive immunostaining for ki-67.

The positivity index for *Ki-67 *was significantly increased with progression from NCT (< 0.1) to ISCC (40.5 ± 11) (*p *= 0.003). *Ki-67 *expression in CINI was significantly higher than in NCT (*p *= 0.01) (Figure 2d & Table 3).

### Expression of cyclins and CDK

*Cyclin D1 *overexpression was detected in CINIII and ISCC cases only. All samples positive for *cyclin D1 *expression were scored (++) except for 6 cases of ISCC that scored (+++). The PI was significantly higher in ISCC than in CINIII (*p *= 0.001). *Cyclin E *expression was significantly increased from CINI (16.7%; 18 ± 1.1) to ISCC (88.4% PI = 97 ± 13.8), (*p *= 0.001). Similarly, *CDK4 *expression was significantly increased from CINI to ISCC (*p *= 0.001) (Figure 1f, 1g & Table 3).

**Figure 3:**
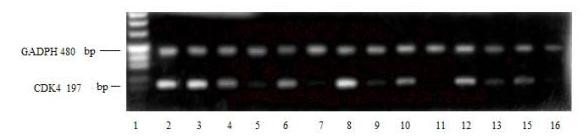
An ethidium bromide-stained 2% agarose gel for *CDK4 *gene amplification, lane 1: molecular weight marker (MW), lanes 2–7: cases of invasive squamous cell carcinoma, lanes 8–12: CIN cases, lanes 13–14: normal cervical tissue samples, lane 15: a negative control. Lanes 2, 3, 8 show *CDK4 *gene amplification.

**Figure 4:**
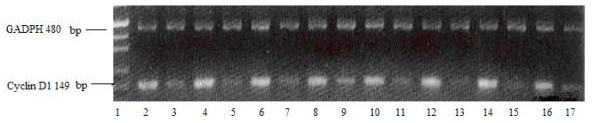
An ethidium bromide-stained 2% agarose gel for *cyclinD1 *gene amplification, lane 1: MW, lanes 2–8: cases of invasive squamous cell carcinoma, lanes 9–14: CIN cases, lanes 15–16: normal cervical tissue samples. Lanes 2,4,6,8,10,12,14 show *cyclinD1 *gene amplification.

**Figure 5:**
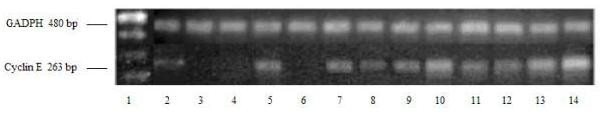
An ethidium bromide-stained 2% agarose gel for *cyclinE *gene amplification, lane 1: MW, lanes 2–4: normal cervical tissue samples, lanes 5–9: CIN cases, lanes 10–14: cases of invasive squamous cell carcinoma. Lanes 10, 13, 14 show *cyclinE *gene amplification.

On the other hand, *cyclin D1 *gene amplification was detected in 20 (46.5%) ISCC and 7 (18.4%) CINIII cases (The concordance between both techniques was 90 %). *Cyclin E *gene amplification was detected in 2 cases (11.1%) of CINI, 5 (45.5%) CINII, 21 (55.3%) CINIII and 38 (88.4%) ISCC (The concordance between both techniques was 97%). *CDK4 *gene amplification was detected in 2 cases of CINI, 4 cases of CINII, 15 cases of CINIII and 39 cases of ISCC (The concordance between both techniques was 96%) (Figures 3, 4, 5).

### Expression of CDKIs

Nuclear immunoreactivity for *p21*^***waf ***^was sporadically detected in NCT, CINI and CINII. However, diffuse staining for *p21*^*waf *^was detected in CINIII and ISCC. There was a significant difference in the PI of *p21*^***waf ***^between CINII and CINIII as well as between CINIII and ISCC (*p *= 0.01) (Figure 2c & Table 3).

*p27*^*Kip*1 ^expression was significantly decreased with disease progression (*p *= 0.003). The protein was found in all NCT and CINI, in 63.6% of CINII, 26.3% of CINIII and in 13.9%of ISCC (Figure 1a–c & Table 3).

*p16*^*INK*4*A *^overexpression was detected in 4 CINII, 24 CINIII and 40 ISCC. The PI was significantly higher in ISCC than in CINIII (*p *= 0.01) (Figure 1d, 1e & table 3). Although abnormal conformers were detected in 3 ISCC cases by SSCP, no mutations were found in any of them by sequencing.

#### p53 gene status

*p53 *overexpression was detected in 18.4% CINIII and 44.2% ISCC cases only (Table 3). All positive CIN cases were scored (++) except for a single case that was scored (+) whereas 11 ISCC cases were scored (+++), 5 were (++) and 3 were (+). There was a significant difference in the PI between CINIII and ISCC (*p *= 0.01) (Figure 2a).

*p53 *gene mutations were detected in 5 (11.6%) cases of ISCC only, 3 of them showed protein overexpression. Four cases showed mutations in exon 7 (one had mutation in codon 240 [AGT → ATT, S → I] and 3 in codon 249 [AGG → AGT, A → S]) and the remaining case showed double mutations in exons 5 (codon 132, 1 bp deletion) and exon 8 (codon 290 [CGC → AGC, S → A]) (Figure 7). None of the normal cervical tissue samples or cases of CINI-III showed *p53 *gene mutations.

### mdm-2 gene status

*mdm-2 *overexpression was detected in CINIII and ISCC cases only. All CINIII cases were scored (++) whereas in ISCC, 17 cases were scored (+++) and 5 were (++). There was a significant difference in the PI between CINIII and ISCC (*p *= 0.01) (Figure 2b & Table 3). *mdm-2 *gene amplification was reported in 9 CINIII cases and in 20 ISCC cases. The concordance between both techniques was 95.1%.

### Rb gene status

**Figure 6:**
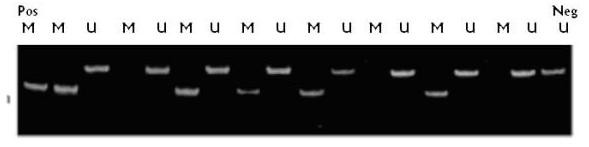
An ethidium bromide-stained 4% agarose gel for *Rb *gene methylation lane 1: methylated control, lane 18: unmethylted control, lanes 8–12: cases of invasive squamous cell carcinoma, lanes 12–17: CIN cases.

**Figure 7:**
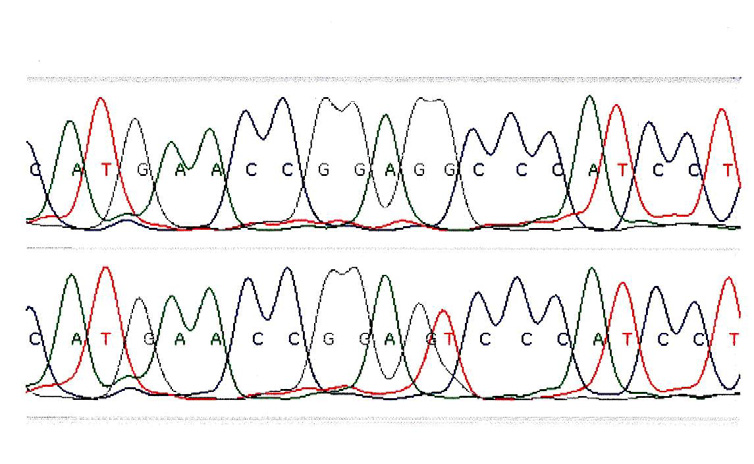
A case of ISCC showing mutation in exon 7 of the *p53 *gene codon 247 by single stranded conformation polymorphism and sequence analysis.

Normal expression of the *Rb *protein was reported in all NCT and CINI, whereas altered expression was detected in 6 (54.5%) CINII cases, 21 (55.2%) CINIII and 29 (67.4%) ISCC. There was a significant difference in the PI between NCT and CINII (*P *= 0.031), CINII and CINIII (*p *= 0.021) and between CINIII and ISCC (*p *= 0.021) (Figure 1h, 1i & Table 3). HZD of the *Rb *gene was found in 20 (46.5%) ISCC cases whereas PM was detected in 15 (39.5%) CINIII and in 26 (60.5%) ISCC (Figure 6). 

### Clinical correlations

There was a significant correlation between altered expression of *Rb *and *p16*^*INK*4*A *^(*p < 0.001*), *p53 *and *mdm2 *overexpression (*p *= 0.003), *cyclin D1 *and *CDK4 *(*p *= 0.001) as well as between increased *cyclin E *and reduced *p27*^*Kip*1 ^expression in all studied groups (*p *= 0.011).

In the group of ISCC, there was significant relationship between *p53*,*mdm2 *and tumor size (*p *= 0.003), and between type (*p *= 0.01), as well as between *p53 *overexpression and lymphovascular invasion, and FIGO stage (*p *= 0.003). Similarly, there was a significant association between reduced *p27*^*Kip*1^, tumor size, tumor type (*p *= 0.047), positive lymph node, stromal invasion (*p *= 0.001&*p *= 0.024) and FIGO stage (*p *= 0.002); between *p21*^*waf *^overexpression and tumor size (*p *= 0.001) and vaginal involvement (*p *= 0.042). Increased *cyclin D1 *was significantly associated with tumor size (*p *= 0.003) and FIGO stage (*p *= 0.008) whereas, increased *cyclin E *expression was significantly associated with tumor size (*p *= 0.001). A high *Ki-67 *index was significantly associated with tumor size, lymph node involvement and FIGO stage (Table 1).

For overall survival on univariate analysis, tumor size (*p *= 0.033), tumor differentiation (*p *= 0.047), lymph node status (*p *= 0.021), FIGO stage (*p *= 0.011), a high *Ki-67 *(*p *= 0.034), *cyclinD1 *overexpression (*p *= 0.011), *p53 *overexpression (*p *= 0.04) and reduced *p27 *(*p *= 0.023) were significant (Table 4). However, multivariate analysis revealed that FIGO stage (RR 4.211, 95% confidence interval [CI] 3.281- 158.21, *p *= 0.003), *Ki-67 *(RR 1.527, 95% confidence interval [CI] 1.025- 20.7, *p *= 0.046) and *cyclin D1 *overexpression (RR 1.296, 95% confidence interval [CI] 3.029- 127.67, *p *= 0.008), *p53 *overexpression (RR 0.296, 95% confidence interval [CI] 3.460- 120.944, *p *= 0.001) and *p27*^*KIP*1 ^loss (RR 3.650, 95% confidence interval [CI] 1.307- 17.67, *p *= 0.014), were significant (Table 5)

## Discussion

The present study is the first to investigate the role of a large panel of cell cycle regulatory genes in Egyptian patients with cervical carcinoma both at the gene and protein levels. The studied population was homogeneous since all patients with CIN and ISCC were positive for HPV16 and/or 18. Tissue samples representing the different stages of transformation of normal cervical epithelium into ISCC have been analyzed for the possible role of the studied markers in etiopathogenesis of cervical carcinoma and their prognostic value.

It has been shown that the E6 and E7 proteins of HR-HPV types disrupt cell cycle checkpoints, particularly affecting *CDKI *linked to the G1- and G2-checkpoints with consequent accumulation of genetic aberrations [19]. To our knowledge, the role of *cyclins*, *CDKs *and *CDKI *in ISCC and its precursor lesions is not well defined yet. Moreover no single study has investigated the contribution of these proteins together and in association with other cell cycle related genes such as *p53, Rb *and *mdm-2*. In the present study aberrant expression of *CDKs *and their inhibitors were detected at several points during the transformation of HPV-infected cervical epithelium.

*Cyclin D1 *amplification and protein overexpression were found in 46.5% and 41.9% of our ISCC cases as well as in18.4% and 13.15% of CINIII cases only. Our results are in agreement with Nicholas et al. [19] and Cheung et al. [15]. In contrast, Bae et al. [20] reported reduced *cyclin D1 *mRNA and protein expression in cases of CIN and ISCC compared to NCT. It is difficult to explain the controversy between our results and those of Bae et al. [20]. However, their study is one of the very few studies showing reduced *cyclin D1 *in cervical carcinoma. Moreover, they reported increased *cyclin D1 *expression with increasing severity of the lesion from high grade squamous intraepithelial neoplasia (HSIL) to ISCC which makes a higher expression in normal epithelium compared to the neoplastic one unlikely.

Our study shows a stepwise increase in the expression level of *CDK4 *from normal to tumor tissues indicating an important role for *CDK4 *at an early stage of transformation of HPV- infected cervical epithelium. Our results confirm the few available reports in this context [15,8]. Although it is well known that *cyclinD1 *is necessary for the activation of *CDK*, the concordance reported in the present study between *CDK4 *and *cyclin D1 *expression was 49% only. We also detected increased *CDK4 *expression in cases of CINI and CINII although these cases did not show simultaneous increase in *cyclin D1*. This could be explained by the involvement of other D-type *cyclins *such as *cyclin D2 and 3 *in the activation of *CDK4 *[21]. Alternatively, the D type *cyclins *may not be required at all for G1 progression in HPV-transformed cervical epithelium since binding of HPV E7 protein to *Rb *leads to release of *E2F *transcription factor omitting the role of *cyclin D1 *in cell cycle progression [19].

Our results denote an important role for *cyclin E *in the early stages of HPV-associated cervical carcinogenesis since protein overexpression and gene amplification were detected during progression from NCT into ISCC. We also noticed a significant association between increased *cyclin E *expression and reduced *p27*^*kip*1^. Our data confirm the results of Dellas et al [21] who demonstrated a feed back inhibitory loop between *cyclin E *and *p27*^*kip*1^. This suggests the presence of a synergistic effect between both genes which will eventually lead to enhanced progression through the cell cycle as a consequence of the proliferative effect induced by increased *cyclin E *expression and the loss of the inhibitory function of *p27*^*kip*1^.

Inactivation of *CDKIs *(*p27*^*kip*1^, *p21*^*waf*^*, p16*^*INK*4*A*^) via reduced expression was reported in various human tumors [22]. In HPV-associated cervical carcinoma, the situation is less clear since some studies showed that the tumor suppressor activity of these proteins is overcome through the action of the viral oncogenes E6/E7 without any change in their expression level [22], others showed that this applies to *p21*^*waf *^and *p27*^*kip*1 ^only whereas *p16*^*INK*4*A *^is usually down regulated [22-24]. Moreover, the HR-HPV types were shown to impair the function but not the expression of the *p21*^*waf *^and *p16*^*INK*4*A *^by rendering them insensitive to *cyclin-CDK *complex formation whereas *p27*^*KIP*1 ^is usually down-regulated [24].

In the present work, a stepwise decrease in *p27*^*kip*1 ^expression and a stepwise increase in *p16*^*INK*4*A *^were found in cervical epithelium as it progressed from normal to a neoplastic one. Our results regarding *p27*^*kip*1 ^expression are in agreement with previously published data [8,24-26]. In contrast, Shiozawa et al., [27] reported in their study a strong *p27*^*kip*1 ^expression in normal cervical epithelia which was markedly reduced to a negligible level in CIN samples. However, in ISCC cases they reported an increased expression of *p27*^*kip*1 ^and demonstrated that the *p27 *protein was bound to cdk2 and cyclin E. Consequently, the authors concluded that, *p27*^*kip*1 ^expression may be involved in the growth regulation of NCT however aberrant function of the *p27*^*kip*1 ^may occur in ISCC of the cervix. Although we can not find a proper explanation for this discrepancy in the results, we assume that the mechanism proposed by Shiozawa et al. [27] might represent an alternative pathway for *p27*^*kip*1 ^inactivation in ISCC of the uterine cervix through binding and sequestration by *cdk2 *and *cyclin E *which render it inactive just as the case with *p53 *and *mdm2*.

In our study, *p16*^*INK*4*A *^overexpression was detected during the early stages of cervical carcinogenesis although no mutation was reported in any of the studied cases. We therefore assume that inactivation of *p16*^*INK*4*A *^gene in HPV-associated ISCC is possibly achieved via mechanism(s) other than gene mutations. Among which is the binding to- and sequestration by other cellular and/or viral proteins. Our results are consistent with Volgareva et al. [22] and Tringler et al. [28] who reported *p16*^*INK*4*A *^overexpression in a high percentage of dysplastic and neoplastic lesions of the cervix uteri. They also mentioned that *p16*^*INK*4*A *^could be used as a surrogate marker for early diagnosis of cervical carcinoma. However, our results regarding *p16*^*INK*4*A *^gene mutations in ISCC contrast with Tripathi et al. [17] who reported *p16*^*INK*4*A *^gene mutation in 15% of their ISCC cases. A difference in sampling methods, clinical and virological features of studied cases or a racial difference could be mentioned as possible explanations for the controversial results between the two studies.

An interesting finding in this study is the significant increase of *p21*^*waf *^which was recognized at CINIII and ISCC cases only. Although it is expected that increased expression of *p21*^*waf *^should suppress the progression of cells in the cell cycle and consequently suppress cell growth, all the cases that revealed *p21*^*waf *^overexpression had a high *Ki-67 *positivity index. Several explanations could be mentioned in this regard including: 1) the interaction between HPV E7 oncoprotein and *p21*^*waf *^which abrogates its inhibitory effect on *cyclin/CDK4 *activities, 2) the occurrence of checkpoint adaptation after constant stimulation, 3) altered or inhibited binding to the *CDK4/cyclin D1 *complex, or 4) mutations in downstream targets of *p21*^*waf *^[3,8,10,28].

Our study is the first to assess *RB *gene HZD and PM in addition to altered protein expression in cervical carcinoma and its premalignant lesions. We detected altered *Rb *expression in the early stages of cervical carcinogenesis whereas PM and HZD were reported in CINIII and ISCC only. Therefore, we assume that altered protein expression in the early stages of neoplastic transformation could be attributed to mechanism(s) other than gene deletion or silencing by PM. Degradation by HPV-E7, sequestration by other cellular proteins or inactivation by yet unidentified mechanism(s) could be mentioned in this context. Our results regarding altered Rb expression are consistent with the few available reports in this context [3,8,28].

Since all ISCC cases are HPV-16 or 18 positive, it is not expected to find accumulation of the *p53 *nor the *p53*-induced proteins in these cases. However, elevated levels of *p53 *and *p21*^*waf *^proteins were seen in a relatively high fraction of cervical cancer together with *mdm-2 *in CINIII and ISCC cases. Our data are comparable to Tsuda et al. [29] and Huang et al. [30] who found *p53 *overexpression in 11.4% and 40.5% of HSIL compared to 56.6% and 30.7% of ISCC cases; respectively. Previous studies on ISCC showed that *p53 *overexpression is not usually associated with gene mutation but with sequestration by cellular or viral proteins of which *mdm-2 *is a highly possible candidate. This is in agreement with our results where *p53 *mutations were detected in 4 cases only whereas mdm*-2 *over-expression was present in 51.2% of ISCC, 13.7% CIN.

In gynecologic oncology, valid prognostic factors are necessary to define biologically similar subgroups for analysis of therapeutic efficiency. In the present study *cyclin D1, p27, p53 *and a high *Ki-67 *showed a prognostic relevance as increased expression of *cyclin D1, p53 and Ki-67 *as well as loss of *p27 *were highly correlated with the standard clinicopathologic prognostic factors for ISCC and they were significantly associated with reduced OS. Our data regarding the prognostic value of *cyclin D1 *and *p53 *are in agreement with Bae et al. [20] and Huang et al. [30], respectively who demonstrated that *cyclin D1 *and *p53 *overexpression are associated with decreased disease free survival (DFS) and OS rates. Similarly, our data regarding *p27 *are consistent with previous reports [16,22] which considered *p27*^*KIP*1 ^an independent prognostic factor that significantly correlates with poor survival in ISCC.

**Figure 8:**
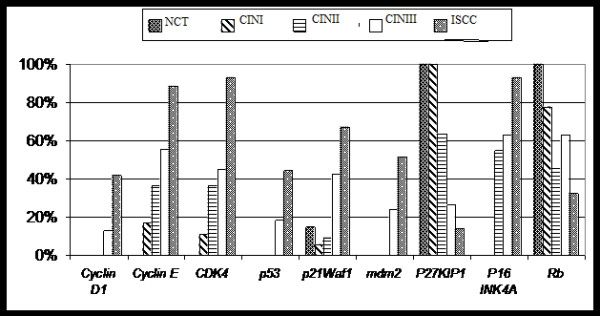
The percentage of protein expression of different studied markers in the studied groups.

We conclude that, infection of cervical mucosa by HPV16 and 18 leads to deregulation of the cell cycle via altering the expression level of certain genes. Whereas *p27*^*KIP*1^, *cyclin E, CDK4*, *p16*^*INK*4*A *^and *Rb *aberrations are early events; *cyclin D1, mdm-2, p53 *and *p21*^waf ^aberrations occur late in cervical carcinogenesis (Figure 8). Together, these alterations lead to acceleration of the cell cycle with increased proliferation rate, as indicated by a high *Ki-67 *PI, and acquisition of more genetic damage. Our results also provide evidence that the application of immunohistochemical tests for *p16*^*INK*4*A*^*, cyclin E, p27*^*KIP*1 ^could help in early diagnosis of cervical carcinoma since alterations affecting the expression level of these proteins occur at an early stage of cervical carcinogenesis. Therefore they could be used as surrogate markers for early detection of ISCC and for monitoring patients with cervical dysplasia. On the other hand, *p53, cyclin D1, p27*^*KIP*1 ^and *Ki-67 *are independent prognostic factors in cervical cancer that could be used for predicting the clinical outcome of those patients.

## Conclusion

It seems that aberrations involving *p27*^*KIP*1^, *cyclin E, CDK4*, *p16*^*INK*4*A *^are early events in HPV 16- and 18-associated cervical carcinoma, whereas *cyclin D1 and p53 *pathway abnormalities are late events. The use of immunohistochemical tests for *p16*^*INK*4*A*^*and cyclin E*, may be of help in early diagnosis of cervical carcinoma. To predict the disease outcome of cervical cancer patients, only FIGO stage *p53, cyclin D1, p27*^*KIP*1 ^and *Ki-67 *are independent prognostic factors.

## Abbreviations

Human papillomavirus (HPV), squamous cell carcinoma (ISCC), Normal cervical tissues (NCT), homozygous deletion (HZD), Promoter Methylation (PM), Overall survival (OS), High risk HPV types (HR-HPV), Carcinoma in situ (CIS), *cyclin dependent kinases (CDKs), cyclin dependent kinases *inhibitors *(CDKIs)*, National Cancer Institute (NCI), World Health Organization (WHO), International Federation of Gynecolology and Obstetrics (FIGO), 3'-3' diaminobenziine (DAB), Phenyl alanine hydroxylase (PAH), High grade squamous intraepithelial neoplasia (HSIL), Large non-keratinized squamous cell carcinoma (LNK-SCC), Large keratinized squamous cell carcinoma (LK-SCC), Small cell carcinoma (SmCC), Positivity index (PI).

## Competing interests

The author(s) declare that they have no competing interests.

## Authors' contributions

**AAB**: She put the study design, she is the pathologist responsible for diagnosis of cases and immunohistochemical studies, she participated in doing the molecular studies and she wrote the manuscript.

**ARNZ**: He participated in the study design, doing the molecular studies and wrote the manuscript and he was responsible for the detection and typing of HPV and coordinated the whole work team.

**MS**: She participated in the study design, participated in doing the molecular studies and in writing the manuscript.

**ML**: He was responsible for the clinical part of the study as well as for the management and follow-up of carcinoma cases.

**MM**: The statistician responsible for the statistical and survival analysis of the manuscript.

**OAS**: The gynecologist responsible for collecting samples, clinical assessment and diagnosis as well as for the follow-up of cases.

## Pre-publication history

The pre-publication history for this paper can be accessed here:



## Figures and Tables

**Table 1:** Clinicopathologic features of invasive squamous cell carcinoma cases in relation to different studied markers.

*Clinical features*	*No*. = *43*	*Cyclin D1* *n *= *18*	*Cyclin E* *n *= *38*	*Cdk-4* *n *= *40*	*P27 *^*KIp*1^ *n *= *37*	*P53* *n *= *19*	*mdm2* *n *= *22*	*P21 *^*waf*^ *n *= *29*	*Rb* *n *= *30*	*P16* *n *= *40*	*Ki-67* *n *= *36*
*Tumor size*											
<*4 cm*	15	2	10	12	9	2	4	7	13	13	10
≤*4 cm*	28	16	28	28	28	17	18	22	27	17	26
		**(*p *= 0.003)**	**(*p *= 0.003)**		**p = 0.047**	**p = 0.003**	**p = 0.003**	**(*p *= 0.001)**			**(*p *= 0.008)**
*Tumor type*											
*LK-SCC*	13	7	9	13	7	1	3	10	12	2	8
*LNK-SCC*	26	10	25	24	26	15	16	17	24	24	24
*SmCC*	4	1	4	3	4	3	3	2	3	4	4
					**p = 0.047**	**p = 0.01**	**p = 0.01**				
*Stromal invasion*											
<*1/2*	15	6	13	15	12	7	7	9	9	14	12
≤*1/2*	28	12	25	25	25	12	15	20	21	26	24
					**p = 0.024**			**p = 0.037**			
*Lymph nodes*											
*Positive*	13	5	9	11	7	2	4	10	7	11	7
*Negative*	30	13	29	29	30	17	18	19	23	29	29
					**p = 0.001**						p = 0.001
*Lympho- vascular*											
*Positive*	7	5	5	5	4	2	2	5	4	5	6
*Negative*	36	13	33	35	33	17	20	24	26	35	30
					**p = 0.035**	**p = 0.003**	**p = 0.001**				
*Vaginal involvement*											
*Positive*	12	6	10	10	7	5	3	8	9	10	11
*Negative*	31	12	28	30	30	14	19	21	21	30	25
								**(*p *= 0.037)**			
*FIGO Stage*											
*I&II*	17	3	15	16	12	2	9	12	13	17	10
*III&IV*	26	15	23	24	25	17	13	17	17	23	26
		**(*p *= 0.008)**			**p = 0.002**	**p = 0.003**					**p = 0.019**

* LNK-SCC: Large non-keratinized squamous cell carcinoma LNK-SCC: Large keratinized squamous cell carcinoma SmCC: Small cell carcinoma

**Table 2:** Summary of specific antisera and immunohistochemistry conditions

***Protein***	***Antibody***	***Dilution & incubation***	***Antigen pretreatment***	***Positive control***	***Stain***
***Cyclin D1***	P2D11F11 Novocastra	1:50	Microwave	Colon cancer	Nuclear
***Cyclin E***	13A3 Novocastra	1:80	Microwave	Breast cancer	Nuclear
***CDK4***	C-22 Santa Cruz Biotechnology	1:300	Microwave	Colon cancer	Nuclear
***P53***	DO7 DAKO	1:50, 2 h	Microwave	Breast cancer	Nuclear
***P21***^*waf*^	SX118, DAKO	1:50, 2 h	Microwave	Epidermis	Nuclear
***Mdm-2***	Monoclonal IF2 Oncogene	1:20, 2 h	Microwave	sarcoma	Nuclear
***P27***	SX53G8, DAKO	1:80, 1 h	None	Breast cancer	Nuclear
***P16***	6H12 Novocastra Lab	1:40	Microwave	Epidermis	Nuclear
***Rb***	Rb1(recognizes phosphorylated & non-phosphorylated protein) DAKO	1:80	Microwave	Retinoblastoma	Nuclear
***Ki-67***	MIB 1, Dako	1:50	Microwave	Breast cancer	Nuclear

**Table 3:** The level of protein expression of different studied markers in normal, dysplastic and neoplastic cervical epithelium detected by immunohistochemistry

Marker	NCT (20)		CINI (n = 18)		CINII (n = 11)		CINIII (n = 38)		ISCC (n = 43)	
	Pos. Cases	PI*	Pos. cases	PI	Pos. cases	PI	Pos. cases	PI	Pos. cases	PI
***Ki-67***	5 (25%)	< 0.1		6.5 ± 0.5		10 ± 0.45		21.6 ± 9		40.5 ± 11
***Cyclin D1***	0(0%)		0(0%)		0 (0%)		5(13.1%)	24.7 ± 3.6	18(41.9%)	63.2 ± 10.8
***Cyclin E***	0(0%)		3(16.7%)	18 ± 1.1	4(36.4%)	30 ± 4.4	21(55.3%)	60 ± 10.2	38(88.4%)	97 ± 13.8
***CDK4***	0(0%)		2(11.1%)	5 ± 0.5	4(36.4%)	12 ± 1.7	17(44.7%)	35 ± 4.6	40(93%)	92 ± 14.7
***p53***	0(0%)		0(0%)		0(0%)		7(18.4%)	44 ± 5.6	19(44.2%)	76 ± 9.1
***p21***^*waf*1^	3(15%)	2.8 ± 0.2	1(5.5%)	7 ± 0.5	1(9.1%)	9.2	16(42.1%)	58 ± 7.1	29(67.4%)	89 ± 10.6
***mdm-2***	0(0%)		0(0%)		0(0%)		9(23.7%)	66 ± 8.2	22(51.2%)	89 ± 10
***p27***^*KIP*1^	20(100%)	90 ± 8.6	18(100%)	77 ± 10.5	7(63.6%)	60 ± 11.7	10(26.3%)	20 ± 5.5	6(13.9%)	9 ± 1.1
***p16***^*INK*4*A*^	0(0%)		0(0%)		4(36.4%)	24 ± 4.7	24(63.1%)	70 ± 12.6	40(93%)	98 ± 12.0
***Rb†***	0	74 ± 11.6	0	68 ± 11.1	6(54.5%)	54 ± 8.6	21(55.2%)	25 ± 1.6	29 (67.4%)	7 ± 1.0

PI = Positivity index

†: Altered expression

**Table 4:** Univariate analysis of the clinicopathological factors and tested markers for overall survival

**Predictive variables**	***P value***
Age (< 50; ≥ 50)	0.919
Tumor type	0.631
Tumor size (< 4 cm, ≥ 4 cm)	**0.033**
Tumor differentiation (well differentiated, poorly differentiated)	**0.047**
Lymph node status (positive, negative)	**0.021**
Stromal invasion (positive, negative)	0.664
Lymphovascular invasion (positive, negative)	0.831
Vaginal involvement (positive, negative)	0.532
FIGO stage (early, late)	**0.011**
	
Cyclin D1	**0.011**
Cyclin E	0.817
CDK4	0.532
p53	**0.040**
mdm-2	0.751
p21^WAF^	0.056
p16^INK4A^	0.855
p27^KIP1^	**0.023**
Rb	0.651
Ki-67	**0.034**

**Table 5:** Multivariate analysis of the clinicopathological factors and tested markers for overall survival

***Predictive variables***	***RR***	***95% CI***	***P value***
Tumor size	3.446	1.107–8.155	0.082
Tumor differentiation	4.073	0.792–20.957	0.093
Lymph nodes	3.921	5.283 – 12.711	0.075
FIGO Stage	4.211	3.281 – 158.21	**0.003**
Cyclin D1	1.296	3.029 – 127.67	**0.008**
p53	0.296	3.460–120.944	**0.001**
p21^waf1^	2.56	1.531–6.015	0.172
p27^KIP1^	3.650	1.307–17.67	**0.014**
Ki-67	1.527	1.025 – 20.7	**0.046**
